# Significant other behavioural responses and patient chronic fatigue syndrome symptom fluctuations in the context of daily life: An experience sampling study

**DOI:** 10.1111/bjhp.12179

**Published:** 2015-12-24

**Authors:** Rebecca Band, Christine Barrowclough, Richard Emsley, Matthew Machin, Alison J. Wearden

**Affiliations:** ^1^ School of Psychological Sciences & Manchester Centre for Health PsychologyUniversity of ManchesterUK; ^2^ Centre for Applications of Health PsychologyUniversity of SouthamptonUK; ^3^ Centre for BiostatisticsInstitute of Population HealthUniversity of ManchesterUK; ^4^ Centre for Health InformaticsInstitute of Population HealthUniversity of ManchesterUK

**Keywords:** chronic fatigue syndrome, Experience Sampling Methodology, significant others, responses

## Abstract

**Objective:**

Significant other responses to patients’ symptoms are important for patient illness outcomes in chronic fatigue syndrome (CFS/ME); negative responses have been associated with increased patient depression, whilst increased disability and fatigue have been associated with solicitous significant other responses. The current study aimed to examine the relationship between significant other responses and patient outcomes within the context of daily life.

**Design:**

Experience Sampling Methodology (ESM).

**Method:**

Twenty‐three patients with CFS/ME and their significant others were recruited from specialist CFS/ME services. Sixty momentary assessments, delivered using individual San Francisco Android Smartphones, were conducted over a period of 6 days. All participants reported on affect, dyadic contact, and significant other responses to the patient. Patients reported on symptom severity, disability, and activity management strategies.

**Results:**

Negative significant other responses were associated with increased patient symptom severity and distress reported at the same momentary assessment; there was evidence of a potentially mediating role of concurrent distress on symptom severity. Patient‐perceived solicitous responses were associated with reduced patient activity and disability reported at the same momentary assessment. Lagged analyses indicate that momentary associations between significant other responses and patient outcomes are largely transitory; significant other responses were not associated with any of the patient outcomes at the subsequent assessment.

**Conclusion:**

The results indicate that significant other responses are important influences on the day‐to‐day experience of CFS/ME. Further research examining patient outcomes in association with specific significant other behavioural responses is warranted and future interventions that target such significant other behaviours may be beneficial.

**Statement of contribution**

***What is already known on this subject?***

The existing literature has identified that significant other responses are important with respect to patient outcomes in CFS/ME. In particular, when examined cross‐sectionally and longitudinally, negative and solicitous significant other responses are associated with poorer illness outcomes. This study is the first to examine the momentary associations between negative and solicitous responses, as reported by the patient and significant other, and patient‐reported outcomes. An ESM paradigm was used to assess these temporal relationships within the context of participants’ daily life.

***What does this study add?***

- Negative responses were associated with increased momentary patient distress and symptoms.
- Perceived solicitousness was associated with activity limitation but less perceived disability.
- The impact of significant other responses on patient outcomes was found to be transitory.

## Background

Chronic fatigue syndrome or myalgic encephalomyelitis (CFS/ME) is a symptomatically defined condition, characterized by severe fatigue and pain (Fukuda *et al*., 1994). Current explanatory models suggest that patients’ cognitive, behavioural, and affective responses to symptoms are important for symptom perpetuation (Deary, Chalder, & Sharpe, 2007; Surawy, Hackmann, Hawton, & Sharpe, 1995). Interpersonal relationships may influence these maintaining factors (e.g., by reinforcing thinking patterns or illness management behaviour, or by providing a source of support or stress for the patient; Band, Barrowclough, & Wearden, 2014; Deary *et al*., 2007), and interactions with significant others have been highlighted as important in the patient illness experience (Dickson, Knussen, & Flowers, 2007). Cognitive behaviour therapy and graded exercise therapy, both of which encourage gradual increases in daily activity levels, are effective treatments for CFS/ME (Castell, Kazantzis, & Moss‐Morris, 2011; White *et al*., 2011). To date, there is little research on the impact that significant others may have on patients’ responses to these treatments.

A recent review of the literature examining significant other responses to CFS/ME identified two types of responses associated with patient‐reported CFS/ME outcomes (Band, Wearden, & Barrowclough, 2015). Significant other negative responses, such expressing frustration at the patient (Kerns & Rosenberg, 1995), have been found to be associated with increased patient depression (Romano, Jensen, Schmaling, Hops, & Buchwald, 2009; White, Lehman, Hemphill, Mandel, & Lehman, 2006). In turn, patient depression has been associated with poorer long‐term patient illness outcomes and responses to treatment (Bentall, Powell, Nye, & Edwards, 2002; Wearden, Dunn, Dowrick, & Morriss, 2012). The review proposed that elevated levels of patient depression may mediate an association between negative significant other responses and increased fatigue (Band *et al*., 2015), and this association has indeed been observed longitudinally (Band *et al*., 2014). The second type of response proposed in the review was solicitous significant other responses, such as encouraging patients to rest or doing tasks on their behalf (Cordingley, Wearden, Appleby, & Fisher, 2001; Kerns & Rosenberg, 1995). In several studies, solicitous responses have been shown to be associated with increased levels of fatigue severity and disability (Brooks, Daglish, & Wearden, 2013; Romano *et al*., 2009; Schmaling, Smith, & Buchwald, 2000) and recently have been linked with poorer patient improvement following cognitive behaviour therapy (CBT; Verspaandonk, Coenders, Bleijenberg, Lobbestael, & Knoop, 2015). The review proposed that these solicitous responses may promote decreased patient activity (Band *et al*., 2015). In turn, reduction in self‐reported activity limitation has been shown to mediate the positive effect of treatment on fatigue (Wearden & Emsley, 2013). However, current understanding of the relationship between significant other responses and patient CFS/ME outcomes is based largely upon cross‐sectional, patient self‐reports of significant others’ responses, and therefore, alternative methodological techniques are required to assess the role of significant other responses in symptom maintenance in CFS/ME.

Experience Sampling Methodology (ESM; Csikszentmihalyi & Larson, 1987) utilizes repeated assessments made within the flow of daily life to assess temporal associations between variables (Myin‐Germeys, Delespaul, & van Os, 2003). As symptoms associated with chronic conditions such as CFS/ME may fluctuate considerably over short periods of time (Prins, van der Meer, & Bleijenberg, 2006), ESM is an appropriate technique to capture relationships between symptom fluctuations and other factors, and also offers the advantage of addressing some of the methodological issues associated with symptom reporting in cross‐sectional research (Redelmeier & Kahneman, 1996; Stone & Broderick, 2007; Stone *et al*., 2003). ESM is also suitable for examining dyadic interactions (Roche, Pincus, Rebar, Conroy, & Ram, 2014), offering potential insight into significant other responses which may vary across contexts within the natural environment (Janicki, Kamarck, Shiffman, & Gwaltney, 2006; Newton‐John & Williams, 2006). Previous studies examining patient‐perceived significant other responses to chronic pain in association with patient pain intensity (Burns *et al*., 2013; Sorbi *et al*., 2006a), and disability (Sorbi *et al*., 2006b) have demonstrated that ESM is a feasible methodology for examining dyadic interactions within chronic conditions such as CFS/ME.

**Figure 1:**
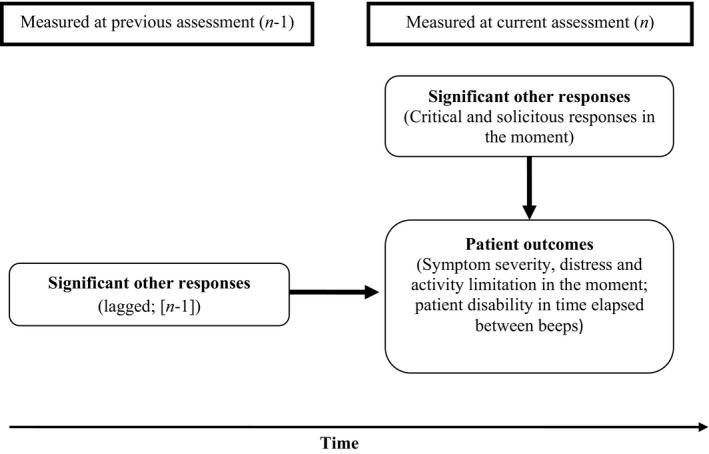
Depiction of the main effects analyses.

The current study used ESM to investigate significant other responses in association with patient‐reported outcomes within the course of daily functioning, by administering matched ESM protocols to patients and their closest significant other, each completing the measures individually and confidentially. It was hypothesized that negative responses would be associated with increased patient distress and symptom severity. In addition, it was predicted that significant other solicitous responses would be associated with increased patient activity limitation and disability. Patient‐perceived and significant other‐reported responses were investigated. Consistent with the cognitive behavioural model, it was predicted that the relationship between patient‐perceived negative significant other responses and symptom severity would be mediated by level of patient distress, whilst the relationship between patient‐perceived solicitous significant other responses and levels of disability would be mediated by patient activity limitation. In addition to exploring associations between responses and patient outcomes at the same momentary assessment, lagged analyses were also conducted, to assess the significant other response reported at the previous momentary assessment in association with change in outcome at the current assessment (Figure 1).

## Method

### Design

The ESM protocol was completed by patients and significant others. In addition, patients completed standardized self‐report outcome measures at the start of the study.

#### ESM sampling schedule, hardware, and software

Participants followed a typical ESM protocol (Myin‐Germeys, van Os, Schwartz, Stone, & Delespaul, 2001); 10 assessments occurred between typical waking hours of 07.30 am and 10.30 pm, for a period of 6 days. A semi‐random sampling schedule was used; each day of ESM sampling was divided in to ten 90‐min periods, and one assessment was made within each throughout the day. Consecutive beeps occurred after a minimum of 15 min and a maximum of 3 hr. To ensure all participants completed assessments within the same time period, access to questions were only available for 15 min. All alerts and ESM data collection were completed on San Francisco Android Smartphones using specialist ClinTouch software (Ainsworth *et al*., 2013). Data entry was completed using a sliding scale on the touch‐sensitive screen (Ainsworth *et al*., 2013).

### Participants

Participants were recruited from UK CFS/ME services and had received a specialist clinical diagnosis of CFS/ME, confirmed against the Oxford Criteria (Sharpe *et al*., 1991). To be eligible, all patients were required to have a willing significant other with whom they lived or had at least 10 hr face‐to‐face contact per week. All participants had to be aged 16 or above, able to provide fully informed consent, and have sufficient English comprehension to complete study measures. Patients were approached to participate at induction to specialist treatment programmes. A total of 22 dyads consented to participate (approximately 10% of patients approached) and one dyad self‐referred into the study, giving a final sample of 23 dyads.

### Measures

#### Non‐ESM Measures

Patients provided demographic information on illness duration, time of diagnosis, and current CFS/ME treatment. In addition, validated and frequently used outcome measures assessing patient fatigue severity and disability were completed prior to the ESM phase of the study.

##### Patient fatigue and disability

Fatigue was measured using the *Chalder Fatigue Questionnaire* (Chalder, Berelowitz, Pawlikowska, Watts, & *et al*., 1993). It consists of 11 items rated on a 4‐point scale (*better than usual – much worse than usual*; total score range 0–33); higher scores indicate greater fatigue severity. The scale has been widely used and well validated in CFS/ME populations (Cella & Chalder, 2010).

The *Work and Social Adjustment Scale* (WSAS; Marks, 1986) measures functioning across five areas: Work, home management, social and private leisure activities, and family relationships. Overall scores are summed from each item (total score range 0–40); higher scores indicate greater disability. The WSAS has been shown to be a reliable, valid tool for assessing disability in CFS/ME (Cella, Sharpe, & Chalder, 2011).

##### Item development

In line with current recommendations, validated non‐ESM measures (Chalder *et al*., 1993; Marks, 1986; Spence, Moss‐Morris, & Chalder, 2005) were used for item development (Palmier‐Claus *et al*., 2011). The items were phrased in language that was familiar to patients; positively and negatively worded items were developed to avoid extreme response bias (Kimhy, Myin‐Germeys, Palmier‐Claus, & Swendsen, 2012). All items were rated on a 7‐point Likert scale anchored with *Not at all* to *A lot* (scored from 1 to 7) and were qualitatively piloted prior to the study commencement to confirm comprehensibility and acceptability. Momentary items began with the phrase ‘Before the beep went off I was…’ or ‘Right now I am…’. STATA (version 11) was used to conduct preliminary analyses assessing the factor structure of the ESM items for individual subscales (Myin‐Germeys *et al*., 2001), using the FACTOR command and the ML method of extraction. The factor solution was determined by identifying the number of factors with an eigenvalue of greater than one, and individual items with a loading of >0.4 were included within the subscale. Internal consistencies of subscales were assessed using the ALPHA command.

*Symptom severity*: Seven items were developed to assess symptom severity ‘in the moment’, that is, at the time immediately preceding the alert, and were completed by patients only. These items reflected core CFS/ME symptoms, including concentration difficulties, termed ‘mental fog’ by UK sufferers. Items were feeling weak, active, tired, well, experiencing pain, experiencing mental fog, and being sleepy. Preliminary principal components analyses indicated that all items loaded on to a one factor solution (α = .79).

*Distress*: Distress was assessed using a single item (‘feeling distressed’), which was included with standard items examining participants’ affect at a momentary level.

*Activity limitation*: Two items were included at the momentary level to assess patient activity limitation. These items were ‘resting to control my symptoms’ and ‘avoiding activities that might make my symptoms worse’, completed by patients only. The alpha coefficient for these items was calculated at α = .80. Whilst it is recognized that Cronbach's alpha is not ideal for testing the reliability of a two item scale, it is likely to produce an underestimate of the true reliability (Eisinga, Grotenhuis, & Pelzer, 2013).

*Disability*: Six items were developed to assess patient disability during the time elapsed since the previous momentary assessment. The phrase ‘Since the last beep I was able to’ preceded items examining household tasks, socializing, leisure activities, leaving the house, work, and general activity; a higher score indicated less disability. All items loaded on to one factor solution (α = .82). These items were completed by patients only.

*Significant other contact and responses*: Participants were asked to report on significant other responses if they indicated dyadic contact at the momentary level. These were completed confidentially by the patient and the significant other on individual smartphones. These items were developed to reflect negative and solicitous responses as defined within the wider literature (Kerns, Turk, & Rudy, 1985; Vaughn & Leff, 1976). Patients and significant others were asked to rate the extent to which the significant other was engaging in the behaviour in the moment before the alert (e.g., ‘Before the beep went off I was doing things for him/her’); responses were rated from ‘Not at all’ to ‘A lot’. Analyses revealed that significant other responses loaded on to a two factor structure; the first factor was labelled as negative responses and included nagging me, irritated with me, and pushing me to do things (α = .92). The second factor was labelled as solicitous responses and included the following behaviours: Doing things for me, looking after me, helping me, and checking up on me (α = .95).

#### Associations with non‐ESM measures

Correlations were conducted between mean levels of ESM reported outcomes (across all valid beeps) with validated non‐ESM outcome measures (Palmier‐Claus *et al*., 2011). Symptom severity reported during the ESM phase was found to be significantly correlated with patient‐reported fatigue severity (Chalder Fatigue scale; *r*s = .567, *p* = .005). High levels of ESM reported disability were found to be correlated with total levels of WSAS disability (*r*s* = *−.538, *p* = .010).

### Procedure

#### ESM briefing

All participants were given a thorough briefing about the study, which included an ESM practice session to ensure that participants understood the meaning of ESM items and ESM rating scales. Researcher and participant contact details were confirmed to maintain contact during the ESM period. Written informed consent was obtained from all participants. Patient non‐ESM measures were completed prior to the ESM phase of the study.

#### Six‐day ESM phase

The ESM phase began on the day following the briefing session and the sampling schedule was synchronized for both patients and significant others. Patients were contacted on Day 2 of the ESM phase to discuss any potential problems and to ensure that participants were happy to continue with the study. Participants were contacted again 2 days later if requested. The ESM phase ended after a period of 6 days.

#### ESM debriefing

After the completion of the ESM phase, all participants were debriefed. All participants were asked to provide evaluative feedback about their experiences of the study.

### Statistical analysis

Experience Sampling Methodology has a hierarchal structure, whereby measures are clustered in three levels: Beeps are nested in days which are nested within participants; therefore, multilevel models were used to test the hypotheses since these take into account the hierarchical structure. The XTMIXED command in Stata (StataCorp, 2009) was used for all continuous outcome variables, with a random intercept for each participant and for each day within participant; betas, 95% CI, and *p*‐values are reported for all associations between independent and dependent variables. On the basis of previous research, preliminary analyses were conducted to examine patient and significant other gender as potential confounders by assessing their relationship with the outcome variables (Ax, 1999; Tamres, Janicki, & Helgeson, 2002). To test the first set of hypotheses, significant other responses were entered as predictor variables into the models with patient outcome variables as the dependent variable for the same momentary assessment (Figure 1). Subsequently, in separate models, lagged significant other responses reported at the previous assessment (*n* − 1) were included as predictors with patient outcomes at the current assessment (*n*) as the dependent variable. Mediation hypotheses were assessed using a difference in coefficients approach. Using the XTMIXED command, we fitted a model without the putative mediator and subsequently including the mediator as a predictor. A change in the coefficient of the main predictor between these two models can be interpreted as evidence of mediation and the size of the indirect effect can be calculated by taking the difference of these coefficients. All analyses were conducted for both significant other‐reported and patient‐perceived responses. Finally, intraclass correlation coefficients (ICCs) were calculated for each of the significant other response variables, to explore the amount of unexplained variation at each level of the model. This allows the proportion of total variability in the outcome to be examined at the person level (i.e., between people), at the day level (i.e., across different days), and at the beep level (i.e., within the same person and day level, but across different beeps).

## Results

### Description of sample

The patient sample (*n* = 23) ranged in age from 17 to 58, with a mean age of 35.5 (*SD *= 13.96) years. Twenty (87%) of the sample were female and 21 (91%) were White British. At the time of recruitment, the median patient illness duration was 5 years (IQR = 10) and 22 (96%) participants were undergoing specialist CFS/ME treatment programmes. Three patients (13%) lived alone but nominated the individual with whom they had the most extensive daily contact as their significant other. Significant others’ (*n* = 23) ages ranged from 19 to 72, with a mean age of 45 (*SD *= 13.35) years old. Thirteen (57%) significant others were female; 11 (48%) were partners, 9 (39%) were parents, and 3 (13%) were daughters of the patient.

#### Patient adherence and retention

None of the 46 participants dropped out before the completion of the study. Three patients (7%) and two significant others (4%) did not complete the level of valid assessments (*n* = 20) traditionally recommended to be retained for analyses (Palmier‐Claus *et al*., 2011). To exploit all of the available data, the analyses presented here were conducted including all participants. Patients completed a mean of 38.74 beeps (*SD *= 14.88), whilst significant others completed a mean of 34.52 beeps (*SD *= 14.93). Patients reported a mean of 18.97 momentary significant other contacts (*SD *= 11.42), and significant others reported a mean of 13.26 (*SD *= 10.90) momentary patient contacts.

### Preliminary analysis

Demographic information relating to the variables included within the multilevel model analyses presented below can be found in Table 1.

**Table 1:** Demographic information for predictor and outcome variables included within the multilevel model analyses

	No of observations	Min, Max	Mean (*SD*) subscale score
Symptom severity	894	0, 41	16.74 (13.19)
Distress	894	0, 7	2.07 (1.64)
Disability	894	0, 7	2.79 (1.46)
Activity limitation	894	0, 7	3.94 (2.09)
Patient‐reported SO solicitous responses	894	0, 7	1.36 (1.84)
Patient‐reported SO negative responses	894	0, 7	0.78 (1.11)
SO reported solicitous responses	809	0, 7	1.00 (1.60)
SO reported negative responses	809	0, 7	0.54 (0.87)

Note

SO = significant other.

#### Confounding variables

Preliminary analyses indicated that neither patient nor significant other gender significantly predicted patient‐reported outcomes on a momentary basis (data not shown). No further analyses including these variables were therefore conducted.

### Effects of negative significant other responses on current patient symptom severity

Both significant other‐reported and patient‐perceived negative responses were associated with increased patient symptom severity reported at the same momentary assessment (Table 2). The negative response variables were then lagged, to reflect the responses reported at the previous beep (*n* − 1), and regression analyses repeated; these indicated that there were no significant associations between significant other negative responses at the previous assessment and current symptom severity.

**Table 2:** The association between significant other responses and patient outcomes in momentary and lagged (*n* − 1) analyses

Predictor variables	Patient distress			Patient symptom severity		
	β	*SE*	*p*	β	*SE*	*p*
Significant other negative responses						
Momentary	**.173**	**.059**	**.003**	**2.295**	**.427**	**.001**
Lagged (*n* − 1)	−.043	.060	.48	.489	.467	.30
Patient‐perceived negative responses						
Momentary	**.188**	.**041**	**<.001**	**.505**	**.149**	**.001**
Lagged (*n* − 1)	.026	.048	.59	621	.357	.082

	Patient activity limitation			Patient disability		
	β	*SE*	*p*	β	*SE*	*p*
Significant other solicitous responses						
Momentary	.046	.053	.38	.034	.031	.27
Lagged (*n* − 1)	.096	.058	.098	−.007	.034	.85
Patient‐perceived solicitous responses						
Momentary	**.080**	**.023**	**<.001**	**.094**	**.038**	**.013**
Lagged (*n* − 1)	.069	.044	.11	.003	.025	.92

*p* < .05 is in boldface.

### The mediating effect of patient distress on the relationship between negative significant other responses and current patient symptom severity

Analyses revealed that negative responses as reported by the patient and significant others were also associated with increased patient distress on a momentary basis. To assess the potential mediating effect of distress, the multilevel model predicting momentary patient symptom severity was recalculated including the potential mediating variable, distress, in the model. When patient distress, significant other‐reported and patient‐perceived negative responses were examined in a single model, only patient momentary distress remained as a significant predictor, suggesting that the effect of significant other negative responses on patient symptom severity is explained by increased levels of momentary patient distress (Table 3).

**Table 3:** Single models combining all predictor variables on patient outcomes at the momentary level

Predictor variables	Patient symptom severity		
	β	*SE*	*p*
Model 1			
Patient distress	**.708**	**.118**	**<.001**
Model 2			
Significant other negative responses	.390	.222	.080
Patient‐perceived negative responses	.165	.185	.37
Patient distress	**.633**	**.139**	**<.001**

	Patient disability		
	β	*SE*	*p*
Model 1			
Activity limitation	**−.196**	**.019**	**<.001**
Model 2			
Patient‐perceived solicitous responses	**.099**	**.022**	**.001**
Activity limitation	**−.204**	**.019**	**<.001**

*p* < .05 is in boldface.

### Effects of solicitous significant other responses on current patient disability

Contrary to study hypotheses, patient‐perceived solicitous responses were associated with *decreased* levels of patient‐reported disability at the concurrent momentary assessment. However, significant other‐reported solicitous responses were not found to be associated with momentary reports of patient disability (Table 2).

### The relationship between activity limitation, solicitous significant other responses, and current patient disability

Increased levels of self‐reported patient activity limitation were significantly associated with higher levels of patient‐perceived solicitous responses at the same momentary assessment but not significant other‐reported solicitous responses. In addition, patient activity limitation was found to significantly predict increased patient disability at the momentary assessment (Table 2). When both variables were included in subsequent multilevel models, patient‐perceived solicitous responses and activity limitation were identified as independent predictors of patient disability on a momentary level (Table 3), indicating that on a momentary basis, patient activity limitation did not appear to mediate the association between solicitous responses and patient disability.

### The variability of patient‐reported outcomes across the different levels of data

The ICC analyses indicate that the majority of the unexplained variation in patient symptom severity and activity limitation occurred at the beep level (i.e., between assessments, within the same patient, and across the same day). However, patient levels of distress and disability indicate much higher levels of variation between individual participants (Table S1). For example, when examining significant other negative responses as the predictor, 66% of the unexplained variation in symptom severity is at the beep level. However, whilst distress also varied most (49%) at the beep level, 35% of the variance was identified at the person level, suggesting that distress varies more between people than symptom severity.

## Discussion

The current study aimed to examine two types of significant other responses hypothesized to be important in association with patient‐reported CFS/ME outcomes within the context of daily life. In line with study hypotheses, significant other negative responses were associated with increases in patient‐reported symptom severity and distress at the same momentary assessment. The relationship between negative significant other responses and increased patient depression has been documented cross‐sectionally (Romano *et al*., 2009; White *et al*., 2006) and patient depression has also been identified as predictive of poorer patient illness and treatment outcomes (Bentall *et al*., 2002; Tamres *et al*., 2002). A recent review of the existing evidence suggested that significant other negative responses may be important for patient illness outcomes by increasing levels of patient depression (Band *et al*., 2015) supported by longitudinal evidence demonstrating the mediational role of increased patient depression between significant other criticism and fatigue severity (Band *et al*., 2014). The findings presented here further support the proposed interpersonal process (Band *et al*., 2015) and extend the current literature by demonstrating the associations between these variables on a momentary basis.

It was also hypothesized that significant other solicitous responses would be associated with increased patient disability and activity limitation. Patient‐perceived solicitous responses were observed to be associated with increased activity limitation at the same momentary assessment, in line with study hypotheses. Previous research has indicated that activity limitation may be an important factor mediating the effect of treatment programmes in reducing fatigue (Wearden & Emsley, 2013), and the current analyses also suggest that patient‐perceived solicitous responses have an independent effect on momentary levels of patient activity limitation. However, the association between patient‐perceived solicitous significant other responses and patient disability was in the opposite direction to that predicted, with increased solicitousness associated with *reduced* patient‐reported disability at the same momentary assessment. This finding would at first sight to appear be inconsistent with the previous literature where, cross‐sectionally, and using different methodologies, increased patient disability and fatigue have been associated with increased significant other solicitous responses (Brooks *et al*., 2013; Romano *et al*., 2009; Schmaling *et al*., 2000). The interpretation of our finding is limited by the correlational associations of these variables. Possibly the finding indicates that when patients perceive themselves as more able to engage in activities such as work and social activities, they also report their significant other as engaging in more solicitous behaviours (e.g., helping or looking after them). Equally, it is also plausible that significant others respond in a more solicitous way when patients are limiting their activity, leading to a perceived reduction in patient disability.

The items developed to assess patient disability showed a significant, moderate correlation with the WSAS measure of disability, suggesting that patients reporting greater levels of momentary disability also reported higher levels of ongoing disability. However, the inconsistency between our findings with respect to solicitous significant other responding and patient disability and those previously reported may reflect the methodological differences between measuring variables within an ESM paradigm and using traditional cross‐sectional questionnaire reports; the ESM data may potentially reflect more state‐like reports of disability, whereas cross‐sectional reports may reflect more stable, global perceptions of disability. ESM data offer the potential to examine temporal relationships between variables as they occur, or between current events (e.g., significant other responses) and subsequent patient outcomes. On a momentary level, patient‐perceived solicitous responses may be experienced as helpful and facilitative by patients, but repeated solicitous exchanges with significant others may impact upon patient outcomes differently over time, as the wider cross‐sectional literature would suggest. To address this possibility, future research examining the impact of significant other behaviour might usefully examine those dyads where the significant other has a persistent and frequent response style (i.e., negative or solicitous) compared with dyads where behavioural interactions with the patient are more variable.

Whilst consistent associations were noted for patient‐perceived and significant other‐reported negative responses, it is worth noting that significant other‐reported solicitous responses were not found to be significantly associated with patient disability or activity limitation, as expected. The inclusion of significant other‐reported responses was beneficial for examining these processes from both a patient and significant other viewpoint, and suggests that the respondent (i.e., patient or significant other) is an important factor that has received little attention in the previous literature.

However, a second, quite different interpretation of our unexpected finding is that solicitous behaviour on the part of significant others actually was leading to reductions in patient disability and that the inconsistency with previous literature is because the solicitousness which we measured was somewhat different from that which has been measured in other studies. Solicitous responding is likely to be a compound construct, and it is possible that some aspects of what might be labelled solicitousness, for example, providing encouragement for appropriate levels of activity, may be helpful for the patient's recovery, whilst other aspects of solicitousness, such as overprotection, may be unhelpful.

Many of the items utilized within the current study were developed specifically, and as a result, it is possible that solicitous response items included within the study do not accurately reflect either the same type or same level of solicitous behaviours typically found to associate with poorer patient outcomes. We further recognize that other constructs utilized within our study, such as distress, are related but not identical to those previously reported in the literature (such as depression; Mead, 2002). Furthermore, the items used to assess activity limitation within the current study required patients to report on their activity levels and make a judgement on why they were limiting their activity, if they were doing so at the momentary assessment. These items limit the interpretation of the associations between solicitous responses, activity limitation, and disability; particularly as we are unable to account for instances where patients may have been resting but were not consciously doing so to control symptoms, for example. Whilst inherently subjective sensations such as fatigue or pain cannot be objectively measured, the development and inclusion in future studies of objective measures such as actigraphy may help overcome some of these difficulties.

There are a number of limitations associated with the current study that need to be acknowledged. As noted above, the temporal associations identified between variables of interest are limited by their correlational nature. Therefore, it is not possible to infer causal relationships from ESM data, and as a result, alternative explanations for these observations must be explored. For example, it is possible that increased patient symptom severity at the momentary level elicited negative significant other responses. The lack of associations between significant other responses and patient outcomes at the subsequent momentary assessment is surprising given the previous literature and suggests that the impact of significant other responses on patient outcomes is, at a momentary level at least, fairly transitory. It is also possible that patient enrolment in specialist CFS/ME treatment programmes prior to entering the study may have impacted upon the relationship between patient illness experiences and significant other responses. Patients may have been at different stages of their treatment programmes; we do not have data regarding the stage of treatment that each patient had reached. Future studies may usefully combine ESM techniques with ongoing treatment programmes to analyse processes of change. Additionally, a final limitation is the reliance upon participant self‐report in generating momentary data, particularly in relation to potentially sensitive questions such as significant other responses. However, confidential electronic data collection ensured that all participants’ data entry remained private and was inaccessible to either participant following assessment completion.

### Conclusions

The novel findings from this study demonstrate that significant other responses are important for patients’ day‐to‐day experience of CFS/ME. In particular, the results indicate that targeting significant other negative responses may be beneficial for reducing temporary increases in patient experience of symptom severity and distress. The association between solicitous responses (as perceived by the patient) and patient activity limitation is important, given the reduction in activity limitation is associated with decreased fatigue severity during patient treatment programmes (Wearden & Emsley, 2013). The results also indicate that perceived helpful responses may be important in facilitating patient perception of increased ability to participate in daily activities. Given that that cognitive behavioural interventions aim to address dysfunctional beliefs about the relationships between symptom experience and activity, it is important to know whether these beliefs and the behaviour patterns they engender are being reinforced by significant others (Wiborg, Knoop, Stulemeijer, Prins, & Bleijenberg, 2010). Future research should seek to examine which specific significant other responses elicit increased levels of patient activity, and which associate most strongly with unfavourable activity management strategies, such as activity limitation. We suggest that it is particularly important to explore further the nature and role of solicitous significant other responding, as this may inform potentially helpful significant other focused interventions.

## Conflict of interest

All authors declare no conflict of interest.

## Supporting information


**Table S1.** Intraclass correlation coefficients (ICC) for potential significant other response predictor variables.
