# Supplementary material for: Significant other behavioural responses and patient chronic fatigue syndrome symptom fluctuations in the context of daily life: An experience sampling study
Source: Br J Health Psychol. 2015 Dec 24;21(3):499–514. doi: 10.1111/bjhp.12179 (PMC4991278; doi:10.1111/bjhp.12179)
Supplement: Supplementary file 1 — Table S1. Intraclass correlation coefficients (ICC) for potential significant other response predictor variables. [file BJHP-21-499-s001.docx]

**Table S1:** Intraclass correlation coefficients (ICC) for potential significant other response predictor variables

| **Model predictors** | | **SO negative responses** | | | **PP negative responses** | | | **Lagged SO negative responses** | | | **Lagged PP negative responses** | | |
| --- | --- | --- | --- | --- | --- | --- | --- | --- | --- | --- | --- | --- | --- |
| **Outcome** | **Level** | **Var** | **SE var** | **ICC** | **Var** | **SE var** | **ICC** | **Var** | **SE var** | **ICC** | **Var** | **SE var** | **ICC** |
| Symptom severity | Person  Day  Beep | 5.26 | .98 | 0.19 | 3.45 | .58 | 0.34 | 5.88 | 1.04 | .23 | 4.11 | .82 | .13 |
|  |  | 4.68 | .60 | 0.15 | 2.19 | .26 | 0.14 | 3.29 | .74 | .07 | 2.76 | .74 | .06 |
|  |  | 9.64 | .26 | 0.66 | 4.30 | .11 | 0.52 | 10.29 | .30 | .70 | 10.24 | .28 | .81 |
| Distress | Person  Day  Beep | .98 | .17 | 0.35 | 1.02 | .17 | 0.37 | 1.03 | .18 | .38 | .98 | .17 | .35 |
|  |  | .67 | .09 | 0.16 | .55 | .07 | 0.11 | .73 | .09 | .19 | .59 | .08 | .13 |
|  |  | 1.15 | .04 | 0.49 | 1.20 | 0.3 | 0.52 | 1.10 | .04 | .43 | 1.20 | .04 | .52 |
|  |  | **SO solicitous responses** | | | **PP solicitous responses** | | | **Lagged SO solicitous responses** | | | **Lagged PP solicitous responses** | | |
| **Outcome** | **Level** | **Var** | **SE var** | **ICC** | **Var** | **SE var** | **ICC** | **Var** | **SE var** | **ICC** | **Var** | **SE var** | **ICC** |
| Disability | Person  Day  Beep | .88 | .15 | 0.36 | .84 | .14 | 0.33 | .86 | .16 | .34 | .85 | .15 | .35 |
|  |  | .59 | .07 | 0.16 | .51 | .06 | 0.12 | .60 | .08 | .16 | .51 | .07 | .13 |
|  |  | 1.01 | .03 | 0.48 | 1.08 | .03 | 0.55 | 1.05 | .04 | .50 | 1.05 | .03 | .52 |
| Activity limitation | Person  Day  Beep | .90 | .18 | 0.18 | .84 | .16 | 0.16 | .86 | .19 | .16 | .84 | .18 | .15 |
|  |  | .68 | .12 | 0.10 | .60 | .10 | 0.08 | .64 | .13 | .09 | .66 | .12 | .09 |
|  |  | 1.79 | .06 | 0.72 | 1.84 | .05 | 0.76 | 1.84 | .06 | .75 | 1.86 | .06 | .76 |

Note: SO = significant other; PP = patient perceived.
